# Explainable Feature Engineering for Multi-Modal Tissue State Monitoring Based on Impedance Spectroscopy

**DOI:** 10.3390/s24165209

**Published:** 2024-08-12

**Authors:** Mahdi Guermazi, Ahmed Yahia Kallel, Olfa Kanoun

**Affiliations:** Chair for Measurement and Sensor Technology, Chemnitz University of Technology, 09126 Chemnitz, Germany; mahdi.guermazi@polytecsousse.tn (M.G.); ahmed-yahia.kallel@etit.tu-chemnitz.de (A.Y.K.)

**Keywords:** bioimpedance spectroscopy, bovine meat, meat freshness, machine learning, F1-score, explainable machine learning, reliable machine learning

## Abstract

One of the most promising approaches to food quality assessments is the use of impedance spectroscopy combined with machine learning. Thereby, feature selection is decisive for a high classification accuracy. Physically based features have particularly significant advantages because they are able to consider prior knowledge and to concentrate the data into pertinent understandable information, building a solid basis for classification. In this study, we aim to identify physically based measurable features for muscle type and freshness classifications of bovine meat based on impedance spectroscopy measurements. We carry out a combined study where features are ranked based on their F1-score, cumulative feature selection, and t-distributed Stochastic Neighbor Embedding (t-SNE). In terms of features, we analyze the characteristic points (CPs) of the impedance spectrum and the model parameters (MPs) obtained by fitting a physical model to the measurements. The results show that either MPs or CPs alone are sufficient for detecting muscle type. Combining capacitance (C) and extracellular resistance (Rex) or the modulus of the characteristic point Z1 and the phase at the characteristic frequency of the beta dispersion (Phi2) leads to accurate separation. In contrast, the detection of freshness is more challenging. It requires more distinct features. We achieved a 90% freshness separation using the MPs describing intracellular resistance (Rin) and capacitance (C). A 95.5% freshness separation was achieved by considering the phase at the end of the beta dispersion (Phi3) and Rin. Including additional features related to muscle type improves the separability of samples; ultimately, a 99.6% separation can be achieved by selecting the appropriate features.

## 1. Introduction

Several meat-related scandals have highlighted the importance of rigorous food quality assessments during food’s production, transport, and storage, especially for consumable proteins such as meat, fish, chicken, and ham, over the past few decades. Exposure to suboptimal temperatures at any stage can lead to the deterioration of biological tissues. Fraudulent practices have also been reported, such as mislabeling older consumable foods. Unethical practices in the meat industry sometimes involve misrepresenting prices by mixing different cuts and selling cheaper cuts under the label of premium cuts, which deceives consumers. This is particularly common with beef, where it can be challenging to directly differentiate between various muscle parts due to their similar appearances. Therefore, developing an efficient system for classifying muscle types and assessing freshness with minimal manual intervention is crucial to ensure accuracy and consistency in their identification. One promising solution to this challenge has been the development of electronic nose (E-Nose) systems [1,2]. These systems typically comprise a sensor array coupled with an intelligent unit that integrates machine learning algorithms for data analysis.

Another reliable method for monitoring meat quality is impedance spectroscopy [3,4,5]. This technique analyzes the electrical properties of meat, specifically its conductivity and permittivity. To automate the extraction of freshness or quality from impedance spectra, the impedance data are used directly (raw) [6,7,8,9] or processed [10,11] before being input into machine learning algorithms. Various features have been explored for the detection of lamb tenderness in [6], including the modulus and phase angle at different frequencies. In [7], a similar approach was proposed to distinguish between deep spoilage and swollen hams, using the frequency range of 100 Hz to 1 MHz. In [10,11], an equivalent circuit model (ECM) was used to model the impedance spectrum, and the resulting parameters were extracted to detect the freshness of bovine and poultry meat. In [10], the training data resulted in a > 99% accuracy, which can be a sign of overfitting, and the relevance of the features was not fully investigated. In [11], the parameters of a Cole–Cole model formed the basis for correlating the sensory attributes and electrical parameters of various types of ham meat from the SM muscle (semi-membranous) and BF muscle (biceps femoris) and reached an accuracy of only 69.2%. Theoretically, the modeling process should have provided a better physical understanding of the phenomena, potentially identifying key features that could improve classification accuracy. However, contrary to expectations, the classification accuracy deteriorated compared to using the raw data. This discrepancy highlights the importance of the careful selection of features for improving model performance.

In this study, we focus on feature selection by investigating the impact of using the same database for two different purposes: accurately determining specific muscle types and assessing the level of freshness. We explore explainable feature engineering for multi-modal tissue state monitoring. To this end, we conduct a study that uses feature importances based on a combined study that uses feature ranking based on F1-score, cumulative feature selection, and and t-Distributed Stochastic Neighbor Embedding (t-SNE) to identify the minimal but sufficient features required to extract freshness and classify muscle type from a dataset containing features from model parameters and characteristic points.

## 2. Materials and Methods

### 2.1. Impedance Spectroscopy Measurements

Impedance spectroscopy measures the complex impedance of meat at different excitation frequencies. This provides us with an interesting way of characterizing the state of the tissue [12,13,14]. Postmortem, biological tissues undergo several changes, including an alteration of their physical properties. These include proteolysis, i.e., the breakdown of protein into polypeptides or amino acids; collagen degradation; and, in many cases, moisture loss [12]. Meat aging significantly affects fiber structure and conductivity, resulting in noticeable changes in complex impedance.

**Figure 1:**
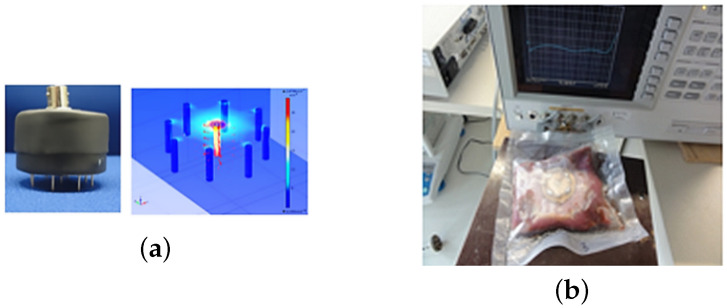
Bioimpedance measurement setup: (**a**) electrodes’ design, (**b**) Agilent 4294 A, together with the examined bovine meat.

In this study, fresh meats were obtained from local farmers in Saxony (Germany). Three distinct muscles have been considered: the Longissimus Dorsi (LD), Rectus Abdominis (RA), and semi-membranous (SM) muscle. Two muscles from each animal source are considered: beef (LDB and RAB) and veal (SMV and LDV). Puncture electrodes were introduced into the meat and kept in place during the whole measurement period, as shown in Figure 1a, within a probe, reducing the influence of anisotropy and establishing good contact [10,15]. The meat was kept in the fridge for two weeks, at a maximum temperature of 4 °C. The measurements were carried out at a room temperature of 25 °C, with the relative humidity of the sealed meat approaching RH 100.

A daily measurement was carried out. The meat was taken out of the fridge and kept at room temperature for one hour before performing impedance spectroscopy measurements (Figure 1). After the measurements, which have a total duration of 10 min, the meat was returned to the fridge. This procedure was repeated for 2 weeks. Starting on the seventh day, the meat showed spoiling and was, therefore, no longer edible. Measurements are carried out in the range of 40 Hz to 110 MHz, mainly in the range of the dispersions α and β [16].

### 2.2. Feature Selection

**Figure 2:**
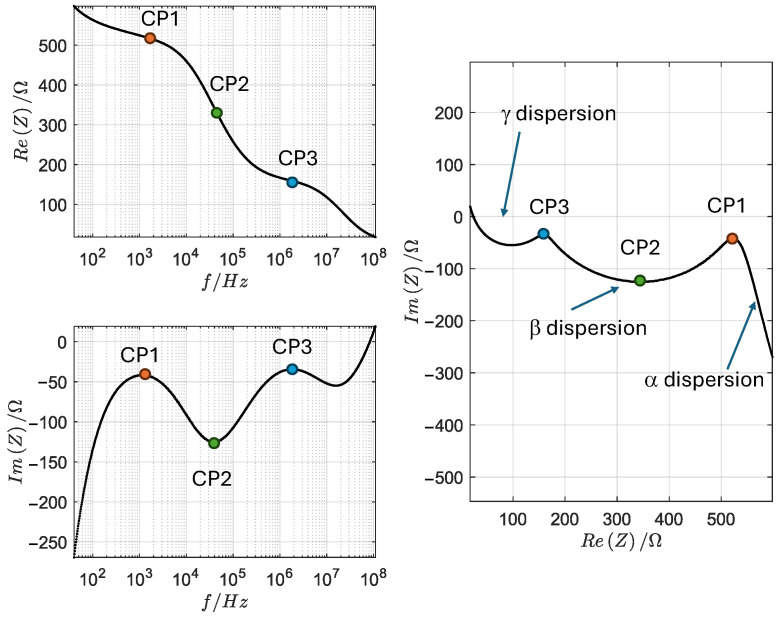
LD beef impedance spectrum in Bode and Nyquist plots with 3 characteristic points (CPs) that separate α,β,andγ regions.

Figure 2 illustrates that the impedance spectrum comprises three distinct dispersion regions: the α, which extends from 40 Hz to ∼10 kHz; the β, which extends from ∼10 kHz to ∼10 MHz; and the β–γ dispersion, which is above 10 MHz. Additionally, the figure demonstrates that the spectra undergo changes as a function of aging. A similar behavior was observed in other muscles. This verifies that the β dispersion is highly susceptible to aging and, therefore, sufficient for this investigation. Despite the difficulty in isolating the dispersion due to the overlap of different dispersions, it is still possible to isolate the region where the β dispersion occurs. This can be achieved by identifying points 1 and 3, which are peak values extracted from the imaginary part of the impedance. These boundary points separate the dispersions into β to α and β to γ, respectively. Point 2 is the characteristic frequency of the β dispersion and is characterized by the minimum value of the imaginary part between the other two points. This process yields three points, which collectively provide 12 pieces of information: their real and imaginary parts, magnitudes, and the phases of their impedance, corresponding to Re1, Re2, Re3, Im1, Im2, Im3, Z1, Z2, Z3, Phi1, Phi2, and Phi3. In a subsequent section, the efficiency of each variable is investigated.

**Figure 3:**
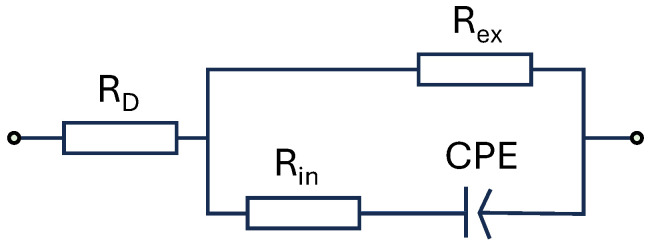
Modified Fricke Model extended by a series resistance.

In this paper, the model parameters are directly derived from the spectrum following the procedure in [5] and subsequently analyzed using the theoretical frameworks proposed by Fricke and the empirical Cole–Cole equation, as indicated in (1): First, the β dispersion is isolated. This is accomplished by picking the frequencies between the peaks of the imaginary values, as shown in Figure 2. Then, the isolated, measured impedance spectrum is fitted based on the Fricke–Cole equation, as shown in Figure 3 and in the following equation:

(1) $$Z_{model}=\left(R_{i}+\frac{1}{{\left(j2\pi f\right)}^{\alpha }.k_{0}}\right)||R_{e}+R_{D}$$

With $R_{e}$: extracellular resistance, $R_{i}$: intracellular resistance, C: the capacitance parameter of a Constant Phase Element (CPE) representing the insulating properties of the cell membrane, α: the exponent of the CPE element, and $R_{D}$: the s Series resistance, including the contact resistance.

**Figure 4:**
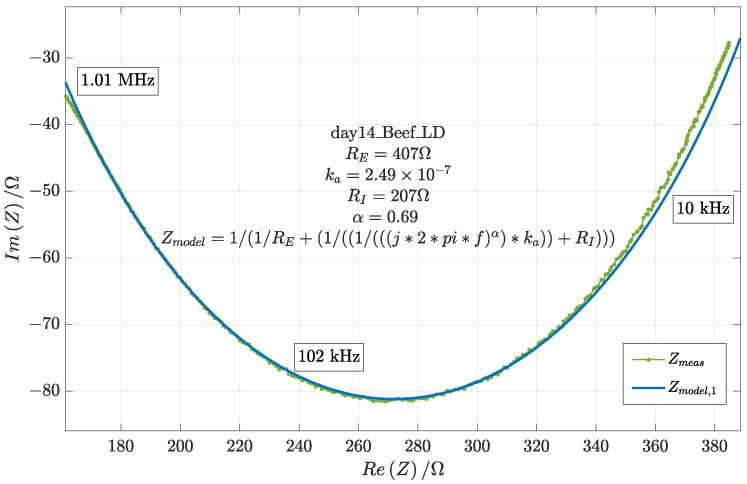
Fitting the Fricke model to the impedance spectrum of the LD Beef muscle measured on day 14.

Using stochastic optimization methods [17], all spectra have been fitted to the model in Figure 3. Exemplary fitting results are shown in Figure 4, which shows a very good fitting result at point 2 in particular. However, a clear deviation can be seen around point 1 and point 3, where an overlap of different dispersions is registered. Nevertheless, since this study focuses on the β dispersion, this deviation is not expected to affect the results.

To better represent the capacitance of the cell membrane, the CPE is converted into a capacitance using the following formula [18]:

(2) $$C=k_{0}\;{\left(\omega_{c}\right)}^{\left(\alpha -1\right)}$$

The angular frequency corresponding to the maximum value of the imaginary part of the impedance in the dispersion area is denoted by β. The CPE is characterized by the quasi-capacitance $k_{0}$ and the phase angle exponent α (not to be confused with the α dispersion). This phase angle exponent, which ranges from 0 (perfect resistor) to 1 (perfect capacitance), quantifies the non-ideality of the capacitance.

Our study’s database comprises 96 measured spectra collected over 13 days (from day 2 to day 14). The meat samples were categorized into two main groups: fresh and non-fresh. The fresh meat category consists of 48 spectra, equally divided between beef and veal (24 spectra each) and representing four different muscles. The remaining 48 spectra correspond to non-fresh meat, with the same composition. The classification of meat as non-fresh was determined through a visual inspection and odor assessment, with measurements from the 8th day onward categorized as non-fresh. From these spectral measurements, we extracted the features listed in Table 1. These features form the basis for the subsequent analysis of meat freshness and muscle type classifications.

To enhance the data, a spline interpolation was employed to represent the spectra between two consecutive days. This was achieved through the implementation of a random number generator in MATLAB between days 1 and 14. Furthermore, random Gaussian noise with a 1% variance was incorporated to reflect measurement deviations and act as jitter information, thereby preventing overfitting. In addition, all the features $d_{i}$ were standardized using the following equation:

(3) $$d_{i,std}=\frac{d_{i}-\mu_{i}}{\sigma_{i}}$$

where $d_{i}$ is the feature in question and one of the features described in Table 1, $\mu_{i}$ is the mean value of the feature $d_{i}$, and $\sigma_{i}$ its standard deviation.

## 3. Explainable Machine Learning for Multi-Modal Classification

In this study, we investigate the potential of determining both meat freshness and muscle type using electrical impedance spectroscopy data. We focus on two distinct objectives:

- Objective 1: Determining muscle type regardless of freshness.
- Objective 2: Determining freshness regardless of muscle type.

### 3.1. Methodology and Feature Analysis

This study begins by removing features with a correlation coefficient greater than 95%. Such features are similar and do not provide additional information for machine learning. The remaining features are then analyzed to assess their contribution to the classification. We employed a 5-fold cross-validation method for model evaluation, partitioning the dataset into five non-overlapping folds. In each of the five folds, 80% of the data was used for training and 20% for va1lidation.

The separability of the classes was evaluated using the average F1-Score of a Support Vector Machine (SVM) [19] classifier with a Radial Basis Function (RBF) kernel for each fold. The SVM-RBF model was chosen for its reliability and popularity in machine learning applications [20]. The F1-score is sensitive to both false positives (e.g., classifying non-fresh meat as fresh) and false negatives (e.g., classifying fresh meat as non-fresh). The F1-score, which balances precision and recall, is calculated as follows:

(4) $$F1=2\cdot \frac{\mathrm{precision}\cdot \mathrm{recall}}{\mathrm{precision}+\mathrm{recall}}=\frac{2\mathrm{TP}}{2\mathrm{TP}+\mathrm{FP}+\mathrm{FN}}$$

Here, TP, FP, and FN represent:

- TP (True Positives): Correctly classified positive instances.
- FP (False Positives): Negative instances incorrectly classified as positive.
- FN (False Negatives): Positive instances incorrectly classified as negative.

Typically, the F1-score ranges from 0 to 1, where 1 indicates the high classifiability, and 0 indicates no classifiability, of the selected features.

To determine the importance of the features, we performed an explanatory model analysis using feature permutation. We systematically changed one piece of information at a time by randomly shuffling its values [21]. We then observed how this change affects the performance of our model, specifically its ability to correctly classify the results, that is, the decrease in the F1-score. Features that cause the largest decreases in the F1-score are considered the most important for classification.

To further validate the importance ranking of the features and determine the optimal feature subset, we performed a cumulative feature selection process. Starting with the highest-ranked feature, we progressively added features based on their importance and evaluated the performance of the model. This approach allows us to identify the minimum number of features that yield the highest precision and recall, thereby optimizing the efficiency and effectiveness of the model. This is carried out to minimize the complexity of the model and its associated training resources (e.g., computational power and training time).

For dimensionality reduction and visualization, we used t-SNE to analyze class separability. This technique maps the high-dimensional data onto a lower-dimensional manifold while preserving local neighborhood structures [22]. t-SNE is particularly useful for revealing global structures, such as clusters, that can help define boundaries between classes. Given an ideal classification scenario where features can effectively separate the classes, t-SNE is expected to generate distinct clusters, where each cluster should correspond to a specific class (e.g., fresh or non-fresh meat, different muscle types).

The combination of these methods provides complementary information about the classification model. The F1-score and cross-validation give us a reliable measure of overall performance and generalizability. Feature permutation importance helps us to understand which features contribute most to the classification task. Cumulative feature selection allows us to optimize the feature set. t-SNE visualization provides insight into the separability of the classes in the feature space, potentially revealing ambiguities in their classification or highlighting particularly effective feature combinations. We configured t-SNE with the following parameters (Table 2).

These parameters were chosen based on recommendations from [22]. The Manhattan (L1) norm is used to measure data similarity. The perplexity, which represents the effective number of neighbors, is set to 30. We set the maximum number of iterations to 1000 and initialize using a Principal Component Analysis (PCA) to ensure reproducibility.

### 3.2. Feature Selection

**Figure 5:**
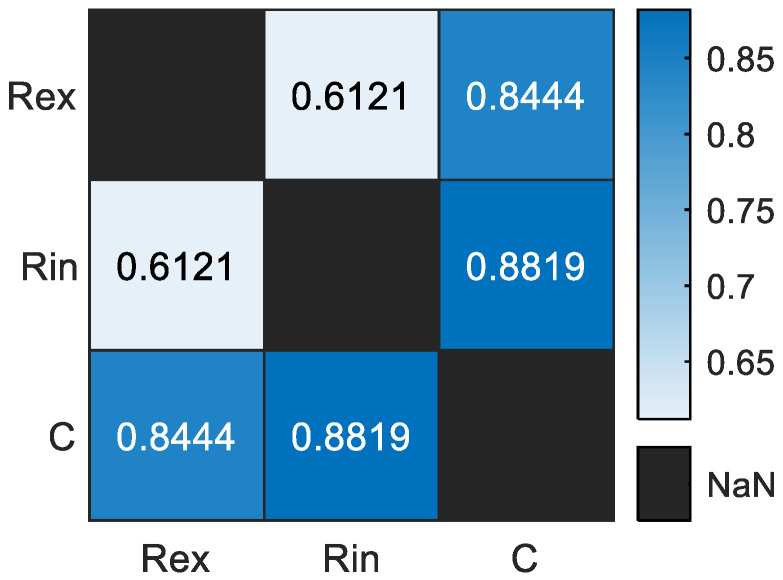
Correlation matrix of the model parameters.

To analyze the MP features, we constructed a correlation matrix (Figure 5). The results show relatively low correlation values, ranging from 0.61 to 0.88, suggesting that these features are suitable for classification.

**Figure 6:**
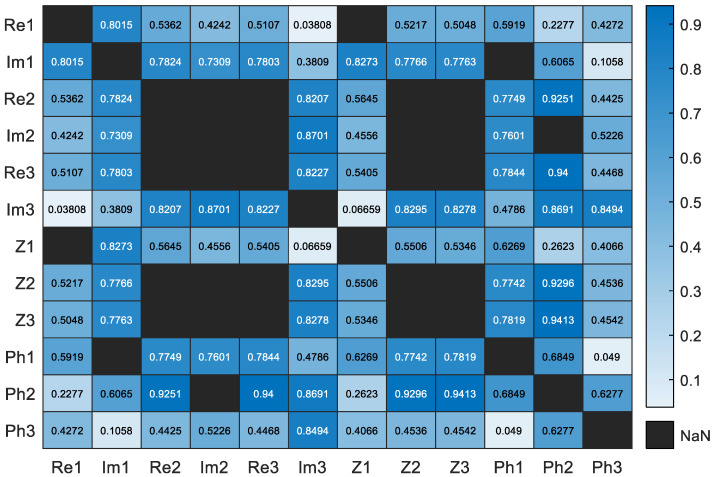
Correlation matrix of characteristic point parameters.

For the CP features, we examined the correlations between different points (Figure 6). The results indicate that the impedance values of the real and imaginary parts are correlated with those of magnitude and phase for points 2 and 3, which correspond to the β and γ dispersions, respectively.

**Figure 7:**
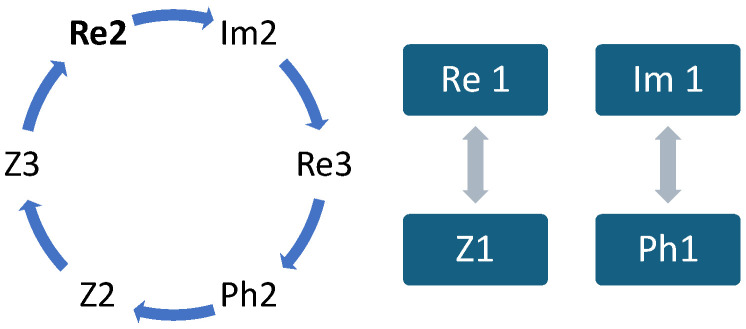
Feature correlation map.

As a result, the rest of this study considers the following five features: Re1, Re2, Im1, Im3, and Phi3. Similarly, the correlation between the properties of the different characteristic points is evaluated. Many features are correlated. A correlation map is drawn in Figure 7 to connect the correlated features.

Next, we evaluate whether MPs, CPs, and a combination of MP plus CP features can evaluate the separability of their corresponding classes.

### 3.3. Muscle Type Detection

#### 3.3.1. Model Parameters

**Figure 8:**
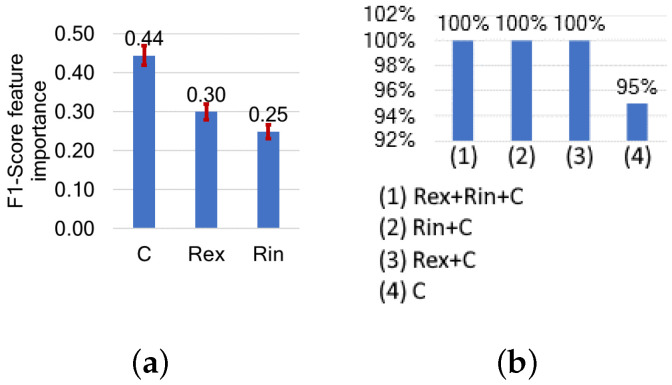
(**a**) Features ranked by their importance, based on their impact on F1-score, and (**b**) F1-scores following cumulative feature selection for muscle detection based on model parameters.

As shown in Figure 8, of the MP features, C has the highest feature importance at 0.44, followed by Rex at 0.3 and Rin at 0.25. Using only C, the F1-score of cumulative feature selection reaches 95%. By combining C with either Rex or Rin, the F1-score increases to 100%, indicating the perfect separability of muscle types. Adding a third parameter does not further improve the results.

**Figure 9:**
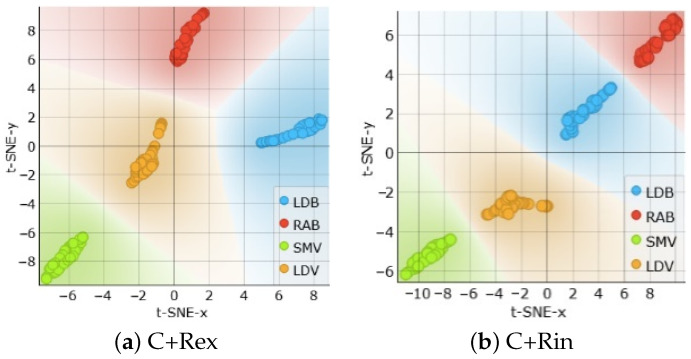
t-SNE sample separability tests for muscle detection using model parameters, based on (**a**) C+Rex and (**b**) C+Rin.

This is further demonstrated using t-SNE in Figure 9a,b. By using C with either Rex or Rin, a separation of the muscle types is possible.

#### 3.3.2. Characteristic Points

**Figure 10:**
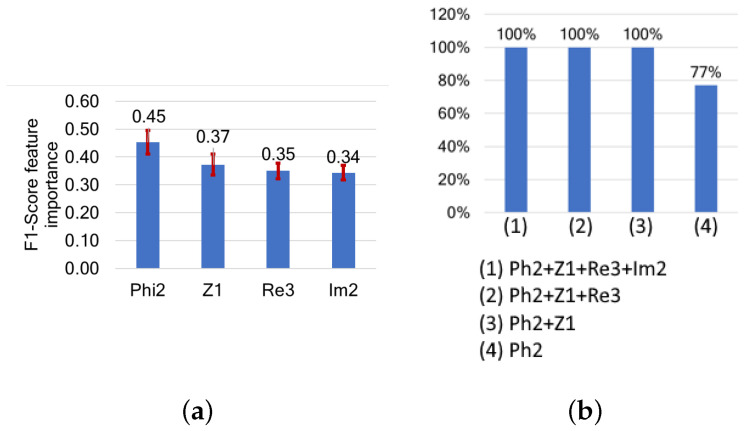
(**a**) Features ranked by their importance based on their impact on F1-score, and (**b**) F1-scores following cumulative feature selection for muscle detection based on characteristic points.

Of the characteristic points, Phi2 (the phase at the characteristic frequency of the β dispersion) is the most influential feature, with an importance score of 0.45. Z1, Re3, and Im2 are also highly influential, with feature importance scores of 0.37, 0.35, and 0.34, respectively. Using only Phi2 to detect the muscle type results in an F1-score of 7%. By including Z1 (the magnitude at the first characteristic point), the F1-score increases to 100%. Similarly, combining Phi2 with either Im2 or Re3 also achieves a 100% F1-score (Figure 10).

**Figure 11:**
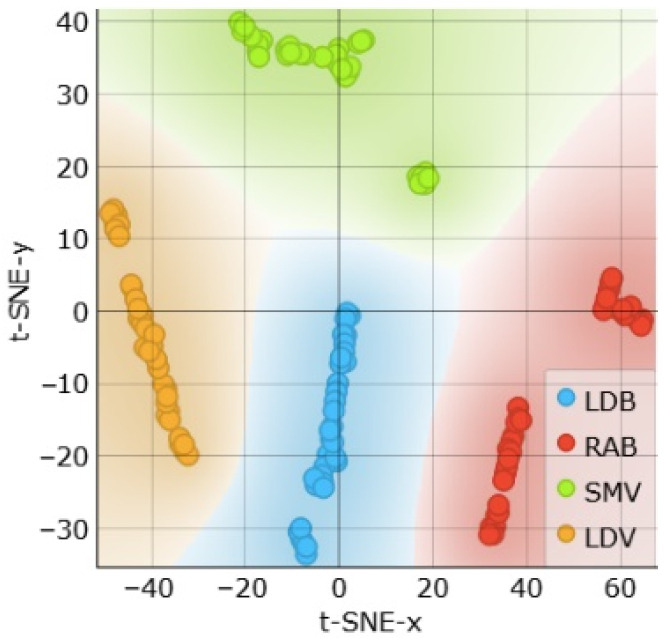
t-SNE sample separability test of muscle detection based on characteristic points.

However, as shown in the t-SNE distribution in Figure 11, this classification is not homogeneous, as each muscle type comprises two separable regions. This suggests that while these features are most influential for muscle detection, they may be capturing additional variations within each muscle type.

#### 3.3.3. Combination of Characteristic Points with Model Parameters

**Figure 12:**
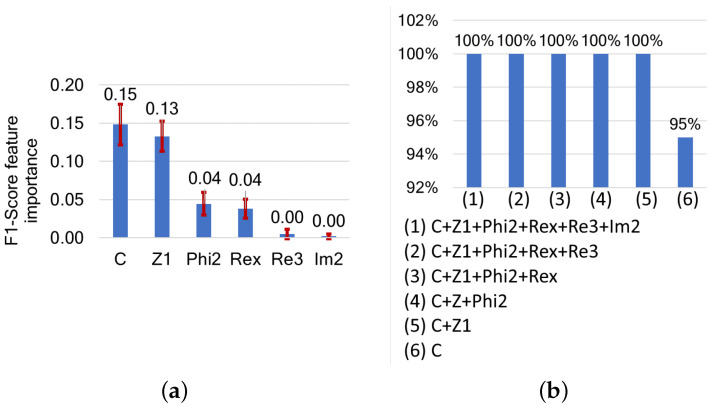
(**a**) Features ranked by their importance based on their impact on F1-score, and (**b**) F1-scores following cumulative feature selection for muscle detection based on combinations of model parameters and characteristic points.

Figure 12 shows a comparison of the efficiency of the combined features from model parameters and characteristic points. Using only the membrane capacitance (C) is sufficient to obtain an F1-score of 95%. When combined with Z1, we achieve 100% accuracy. Adding more features does not further improve the results. From a feature importance perspective, C and Z1 are the most important, both with an F1-score drop of around 0.15, followed by Phi2 and Rex at around 0.04.

**Figure 13:**
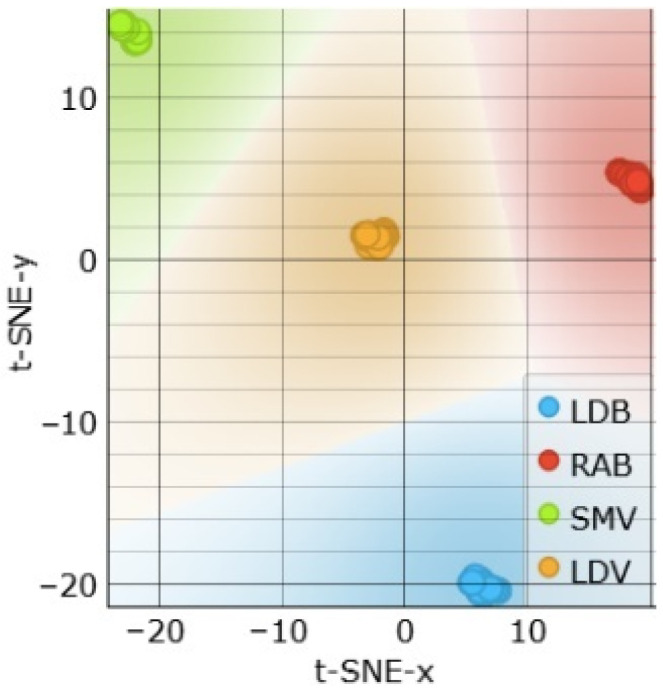
t-SNE sample separability test for muscle detection using Z1 and C features.

This raises the question of whether the combination of C and Z1 could be sufficient for classification and could bring better results than using either model parameters or characteristic points alone. The t-SNE visualization of C+Z1 in Figure 13 shows that the features are very well clusterable, and the muscle types are separable. Unlike in the case of characteristic points alone, t-SNE shows homogeneous feature distribution in this case, contrasting with the results shown in Figure 11.

### 3.4. Freshness Assessment

#### 3.4.1. Model Parameters

**Figure 14:**
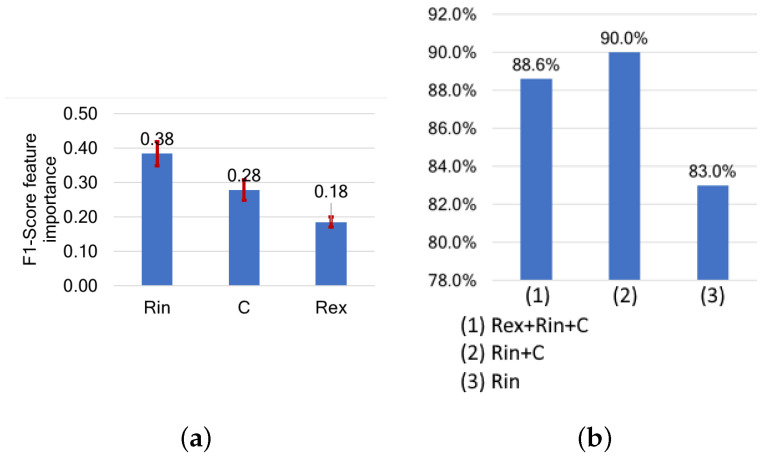
(**a**) Features ranked by their importance based on their impact on F1-score, and (**b**) F1-scores following cumulative feature selection for freshness assessments based on model parameters.

In terms of the MP features, all parameters (Rin, C, and Rex) have similar feature importances, with Rin having the highest at 0.38, followed by C and Rex at around 0.28. Using Rin as the only feature yields an F1-score of 83%, which increases to 90% when combined with C. However, using Rin and Rex results in a decreased F1-score of 82%. The combination of all three parameters (Rin+Rex+C) achieves an F1-score of around 89%. Therefore, the membrane cell capacitance (C) and the intracellular resistance (Rin) appear to be the best features for freshness detection, as shown in Figure 14.

**Figure 15:**
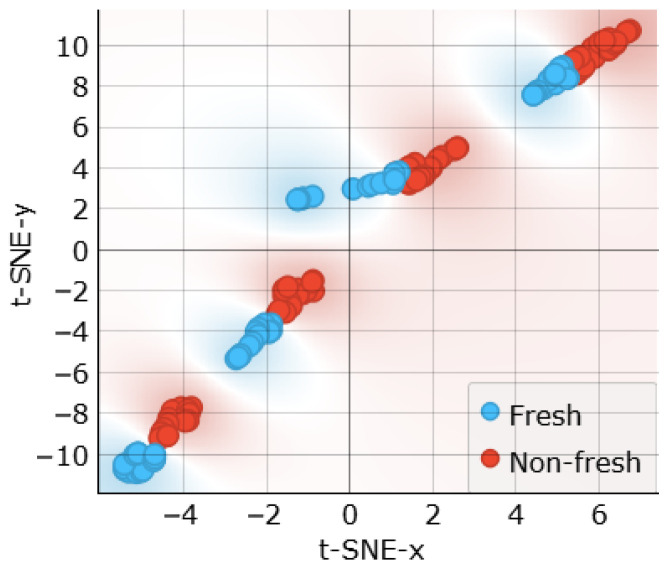
t-SNE sample separability test for freshness assessments using Rin and C features.

The t-SNE visualization in Figure 15 shows that the clusters form around muscle types rather than freshness levels. This suggests that the Rin+C combination is more suitable for muscle type classifications, as discussed in the previous section.

#### 3.4.2. Characteristic Points

**Figure 16:**
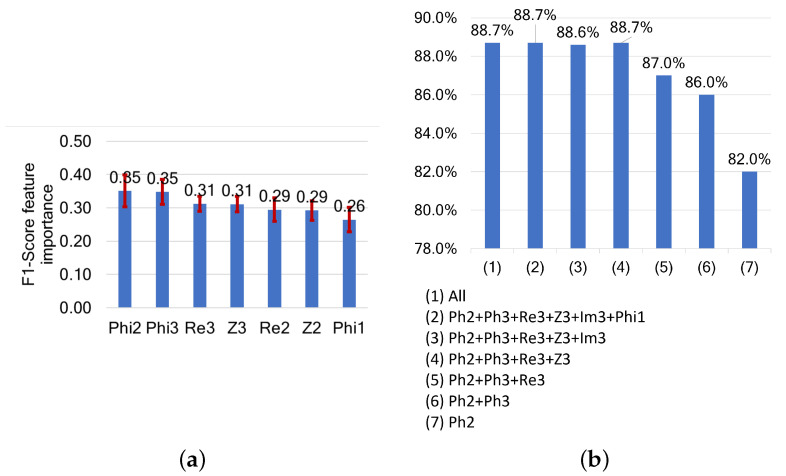
(**a**) Features ranked by their importance based on their impact on F1-score, and (**b**) F1-scores following cumulative feature selection for freshness assessments based on characteristic points.

CPs show behavior similar to that of MPs (see Figure 16). The most influential parameters are Phi2 and Phi3, with a feature importance of around 0.35, followed by Re3 and Z3, with a feature importance of around 0.31.

Using only Phi2 leads to an F1-score of around 82%. This increases to 86% by adding Phi3 and to 87% by adding Re3. The best F1-score of 88.7% is achieved using four features: Phi2, Phi3, Re3, and Z3. Adding more features does not improve the results, with the maximum F1-score remaining around 88.6%.

**Figure 17:**
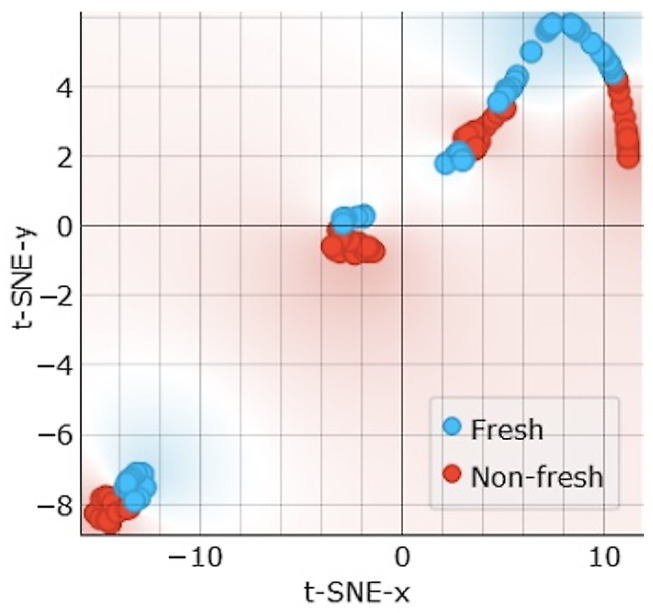
t-SNE sample separability test for freshness assessments using characteristic point features.

The t-SNE plot in Figure 17 shows that the “fresh” and “non-fresh” classes are not clearly separable. This suggests that the selected features (Phi2, Phi3, Re3, and Z3) are not optimal for separating freshness classes but may be more suitable for distinguishing muscle types.

#### 3.4.3. Combined Characteristic Points and Model Parameters

**Figure 18:**
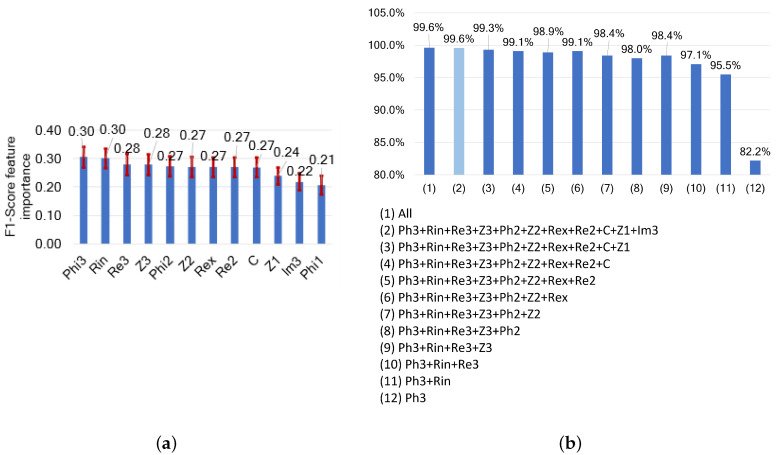
(**a**) Features ranked by their importance based on their impact on F1-score, and (**b**) F1-scores following cumulative feature selection for freshness assessments based on combined model parameter and characteristic point features.

When the MPs and CPs are combined, the results show that Phi3, Rin, Re3, and Z3 are the most influential features. The range of influence of all variables is very narrow, as shown in Figure 18, indicating that all variables contribute almost equally to the freshness classification. Using the F1-score, it is shown that the combination of Phi3 and Rin contributes to an F1-score of 95.5%. Adding more features generally contributes to a better classification and a better F1-score. The highest accuracy is found when all variables are used or when all variables except Phi1 are used, with an F1-score of 99.6%.

**Figure 19:**
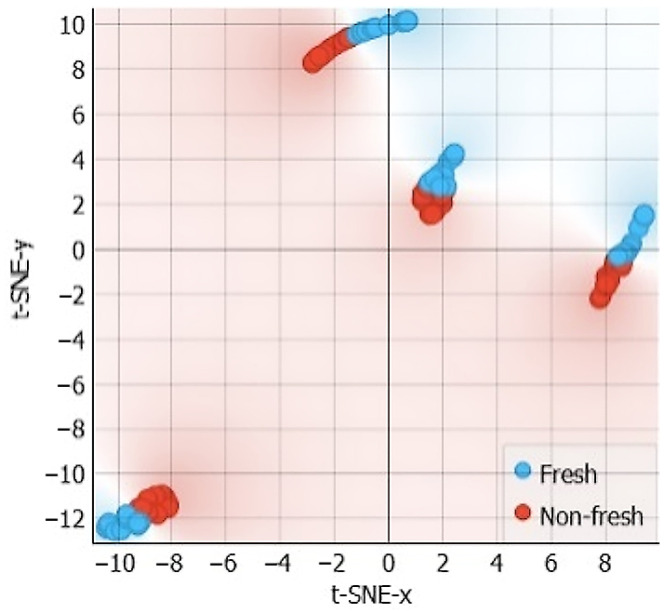
t-SNE sample separability test for freshness assessments using combined model parameter and characteristic point features.

The visualization of t-SNE in Figure 19 shows that a better distribution is possible when combined information is used rather than using only CP or MP features. In the upper right quadrant, the freshness of the LDB, RAB, and LDV muscles can be separated, albeit with a thin line. The SMV muscles, shown in the lower left quadrant, also show separable levels of freshness. This suggests that to effectively separate freshness levels, it is imperative to include features that separate muscle types. In other words, from the combined t-SNE and F1-scores, it is concluded that the muscle type must be implicitly (e.g., through features) or explicitly revealed to provide a higher precision in the freshness assessment.

## 4. Discussion

**Figure 20:**
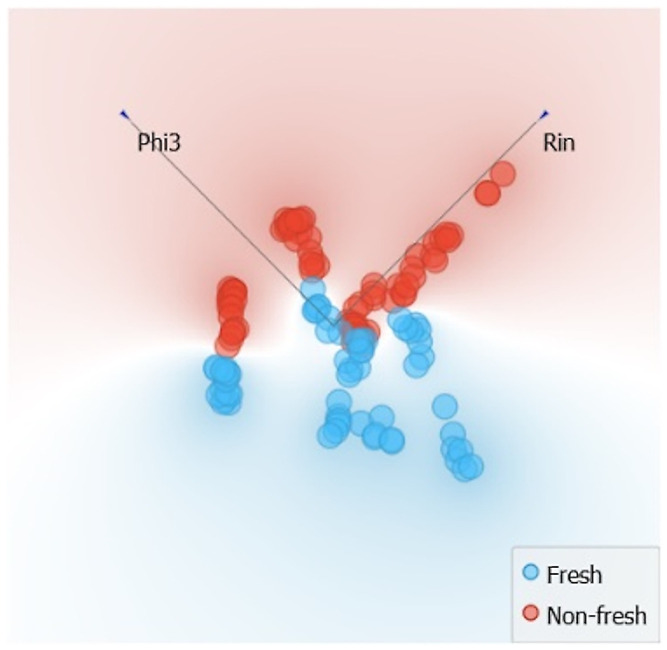
Phi3, C feature projection for sample freshness separability. The arrows indicate the direction of aging.

Examining the results of combined MP+CP features (Phi3, Rex, Rin, and Z3) shows that freshness can be detected with an average F1-score of 99.6%. However, the t-SNE visualization indicates that absolute separability is not achievable, that a classification error margin is expected, and that the use of inferred information about muscle type helps minimize this classification error. For example, the use of Rin+Phi3, as shown in Figure 20, can separate freshness levels, although with minor inconsistencies due to the influence of muscle type.

**Figure 21:**
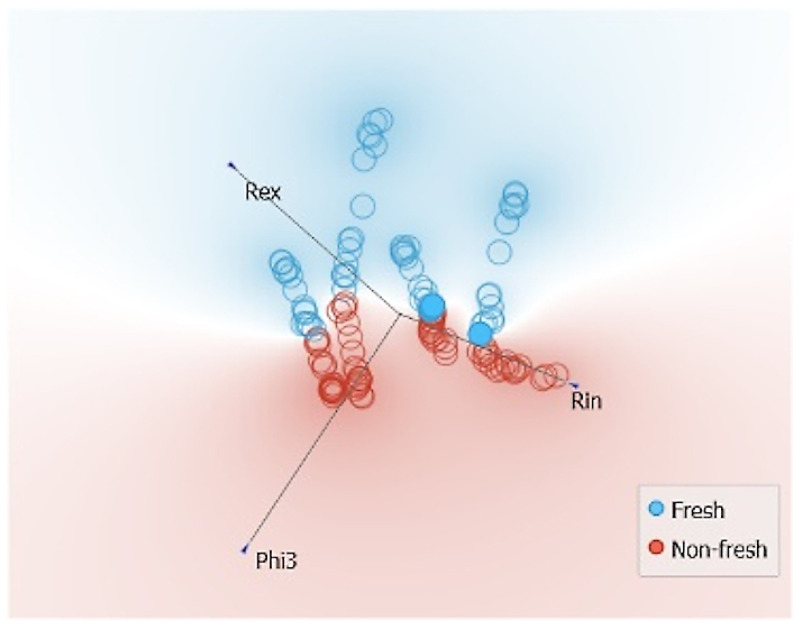
Rin, C, and Rex feature projection for sample freshness separability. The arrows indicate the direction of aging.

This separation becomes more visible by adding a feature such as Rex, as shown in Figure 21. However, these additional features contribute to the implicit differentiation of the muscle type rather than to the freshness classification directly.

The classification accuracy of the SVM model based on only Rin and Phi3 reaches an overall accuracy of 95.6%, with 92.6% accuracy for “non-fresh” and 100% for “fresh” samples. A total of 7.4% of actual fresh samples are misclassified as “non-fresh”. Using all features decreases this confusion to 0.8%, while the model’s accuracy in identifying “non-fresh” samples increases to 99.2%, as shown in Table 3. However, this also means that the features are more tailored to muscle types.

## 5. Conclusions

In machine learning, feature selection is of great importance for concentrating on the measurement data with the most pertinent information before classification. This study concentrated on feature selection and investigated the impact of utilizing model parameters, characteristic points, or their combination for classifying meat type and freshness. A comprehensive analysis was conducted, combining feature importance based on F1-score, score drops, and t-SNE in order to identify the minimal sufficient features for classification. The selection of appropriate explainable engineering features for multi-modal tissue state monitoring is crucial for achieving optimal classifications. The results indicate that either MPs or CPs are sufficient for muscle type detection. When a minimal number of relevant features is required, the combination of cell capacitance (C) and the first characteristic point’s magnitude (Z1) provide a superior solution, leading to enhanced certainty in the classification process and the improved separation of features, with at least 95% accuracy. The classification of freshness proved to be more ambiguous. Based on Rin and C, a freshness classification accuracy of 90% can be achieved. However, the use of all CP features resulted in a reduction in the classification accuracy to 88.7%. By combining the beta phase’s Phi3 with Rin, the freshness classification accuracy reached 95.5%. Freshness is highly correlated with muscle type, and using muscle type information (e.g., C and Z1) can increase freshness classifications’ accuracy, ultimately reaching a separability of 99.6% when selected features are included, leading to improved classifications in meat quality assessments.

## Figures and Tables

**Table 1:** Feature types and their corresponding features.

Feature Type	Feature
Characteristics Points (CPs)	Real parts: Re1, Re2, Re3 Imaginary parts: Im1, Im2, Im3 Magnitude: Z1, Z2, Z3 Phases: Phi1, Phi2, Phi3
Equivalent circuit model parameters (MPs)	Rex Rin C

**Table 2:** Selected t-SNE parameters.

Distance Metric	Perplexity	Max iterations	Initialization
Manhattan (L1)	30	1000	PCA

**Table 3:** SVM classification accuracy.

		Predicted		
		Fresh	Non-Fresh	∑
Actual	Fresh	100.00%	0.8%	200
	Non-fresh	0.00%	99.2%	250
	∑	194	256	450

## Data Availability

The data presented in this study are available on request from the corresponding author.
